# Bodily illusion enhances subjective fear of safety-margin violations surrounding the bodily self

**DOI:** 10.1038/s41598-025-95976-x

**Published:** 2025-04-01

**Authors:** Ryu Ohata, H. Henrik Ehrsson

**Affiliations:** 1 Department of Neuroscience, Karolinska Institutet, Stockholm, Sweden; 2 Human Informatics and Interaction Research Institute, National Institute of Advanced Industrial Science and Technology (AIST), Tsukuba, Japan

**Keywords:** Human behaviour, Sensory processing

## Abstract

All living organisms, including humans, must flexibly adjust their defensive behaviors according to the spatial proximity of potential threats. Previous research has examined fear responses to proximal threats by manipulating distance to the body in simulated 2D or 3D environments; however, the functional role of the sense of bodily self in the interplay between fear and threat proximity remains unclear. Here, we investigated fear responses to proximal and distal fear-relevant stimuli while manipulating the sense of body ownership over a mannequin using a perceptual full-body illusion. Our findings revealed that subjective fear was more sensitive to stimulus location when participants experienced illusory ownership over the mannequin. Furthermore, we observed a significant positive correlation between illusion strength and subjective fear ratings. These findings highlight two aspects of how the sense of bodily self influences human fear experiences: enhancing sensitivity to margin-of-safety violations and amplifying anxiety in response to threat appearance.

**Supplementary Information:**

The online version contains supplementary material available at 10.1038/s41598-025-95976-x.

## Introduction

All living organisms, including humans, must flexibly adapt their defensive behaviors based on the distance between their body and potential threats, such as predators or venomous animals. The threat imminence continuum is a theoretical framework describing the evolutionarily conserved strategy of switching defensive behaviors according to the imminence of threats^1,2^. This framework identifies three stages of perceived levels of threat: pre-encounter, post-encounter, and circa-strike. The primary distinction between the post-encounter and circa-strike stages lies in the likelihood of direct physical contact with the threat. Post-encounter fear is triggered by detecting a distant threat, whereas circa-strike fear emerges when the threat is in close proximity, increasing the probability of a direct predatory attack. In line with this theoretical framework, previous studies have provided empirical evidence that the space immediately surrounding the body, known as peripersonal space (PPS), has ecological and evolutionary significance for possible direct interaction with threats^3–6^. Both humans and nonhuman primates allocate significant attention to PPS. For example, de Haan et al. (2016)^7^ demonstrated that multisensory visuotactile interactions were enhanced when an approaching spider, rather than a receding spider or an approaching butterfly, was proximal to participants’ hand, particularly among individuals who were afraid of spiders. Such facilitation of sensory processing within the PPS may serve an adaptive function, enabling the rapid detection of threat intrusions and the immediate initiation of active defensive responses^8–10^. Thus, the brain continuously monitors the spatial relationship between potential threats and the body, producing corresponding emotional defensive reactions.

Earlier research in human psychology and neuroimaging has investigated fear responses evoked by the spatial proximity of threats by manipulating the distance to fear-relevant stimuli on a 2D computer screen^11–13^. While this approach has provided valuable insights into human fear responses, the low ecological validity of the 2D environment limits the generalizability of these findings because participants were required to mentally simulate threat proximity. To address this limitation, recent studies have employed virtual reality (VR) to simulate a realistic 3D environment^14–18^. VR offers a rich visual experience that immerses participants in a 3D space, enhancing their emotional responses to proximal threats. For example, Rosén et al. (2019)^15^ demonstrated that spatial proximity significantly increased participants’ skin conductance responses (SCRs) when threat stimuli were presented in an immersive 3D environment compared with a 2D computer screen. Thus, immersive paradigms effectively replicate the innate human fear triggered by the invasion of the PPS. However, a fundamental question remains: How do humans perceive threats as being in proximity to their body and experience fear in a virtually simulated environment where a physical body is not actually at risk?

The perception of one’s own body is a fundamental element of human subjective experience. Perceptual awareness of being bodily situated at the center of the perceptual world, known as the sense of bodily self, forms the core of self-consciousness^19–21^. Although the self is closely linked to the physical body in most everyday situations, perceptual full-body illusions can exaggerate the distinction between bodily perception and the physical body’s actual state^22^. These illusions induce an illusory feeling of body ownership over an artificial body^19,23^. A full-body ownership illusion (also called ‘body swap illusion’ or ‘body transfer illusion’) is elicited when a participant views a mannequin (or virtual body) from a natural (first-person) perspective through a head-mounted display (HMD) and sees it being touched while simultaneously feeling synchronous touches on corresponding parts of their own body. The correlated visual and somatosensory cues create the illusory perception that what one sees and what one feels are the same, leading participants to perceive the mannequin’s body as their own and experience the touches as originating from the artificial body. This sense of body ownership depends on the integration of visual, tactile, proprioceptive, and other bodily signals, governed by principles of spatiotemporal congruence, sensory uncertainty, and perceptual priors^23–25^. This phenomenon is a whole-body version of the classic rubber hand illusion^26–30^. Importantly, full-body illusions also alter the perceived location of the self in space, making participants feel as though their selfhood has been relocated to the position of the artificial body^31–35^. Noel et al. (2015)^36^ demonstrated that a full-body illusion can shift the boundaries of PPS from the participant’s actual physical location toward the perceived location of their bodily self. This line of research highlights that changes in the space where we experience the bodily self also modify the spatial relationship between oneself and the surrounding environment^37–39^. On this basis, we hypothesize that humans perceive threats as being proximal or distal relative to the perceived location of the bodily self rather than the physical body alone; this would result in fear responses that are sensitive to threat distance from an illusory-owned artificial body, even in a virtual environment. In line with this hypothesis, previous studies have shown that an egocentric perspective and a sense of full-body ownership enhance the perception of threatening and nonthreatening approaching visual stimuli, suggesting a link between bodily self-perception and fear response to physically threatening stimuli^23,31,34,40–42^. Here, we target the link with responses to a fear-evoking stimulus at varying spatial distances.

To test this hypothesis, we aimed to characterize the functional role of the sense of bodily self in the interplay between fear and threat proximity. In Experiment 1, we investigated whether a perceptual full-body ownership illusion modulates subjective and physiological fear responses evoked by fear-relevant visual stimuli (spiders) located at proximal and distal locations relative to the self. In Experiment 2, we aimed to examine how subjective fear changes as a function of five distinct distances of fear-relevant visual stimuli from the bodily self. In the two experiments, participants were exposed to spider stimuli or to fear-irrelevant, emotionally more neutral stimuli (butterflies) presented at varying spatial proximities relative to a mannequin’s body, while their sense of body ownership over the mannequin was manipulated using the full-body illusion. We found that subjective fear ratings were more sensitive to the spatial proximity of the fear-relevant stimulus when participants experienced illusory ownership of the mannequin’s body than when they did not experience the bodily illusion under the control condition. We also observed a significant correlation between the strength of illusory experience and the levels of participants’ reported fear regardless of whether the fear-relevant stimuli were located proximally or distally relative to the mannequin. Together, these findings highlight two aspects of how the sense of bodily self influences human fear experiences: (1) enhancing sensitivity to margin-of-safety violations and (2) amplifying anxiety in response to threat appearance.

## Methods

### Apparatus and stimuli

We conducted two distinct experiments (Experiments 1 and 2). In the tasks, the participants viewed prerecorded videos shown on the screen of an HMD unit (Oculus Rift DK2, Oculus VR, Menlo Park, CA, USA). These videos provided a stereoscopic, first-person perspective of a mannequin. The videos had previously been recorded using two identical action cameras (GoPro Hero 7, GoPro Inc., San Mateo, CA, USA) mounted side-by-side on a tripod to simulate the spatial arrangement of human eyes and achieve stereoscopic vision. The 3D videos depicted the mannequin’s body parts (abdomen and left and right upper legs) being stroked with a white Styrofoam ball (6.5 cm in diameter) attached to a 1-m-long wooden rod. The footage was edited using Final Cut Pro X 10.4.7. The left and right camera footage was positioned side by side to occupy the left and right halves of the HMD screen (resolution: 1920 × 1080 pixels). The footage was then segmented into distinct video elements and concatenated according to the task timeline. We superimposed 3D animations of spiders and butterflies over the video elements. Finally, an audio track was added to the concatenated video to provide only the experimenter with instructions of precise timing and duration cues for the stroking through headphones.

We used 3D animations of spiders and butterflies as fear-relevant and fear-neutral stimuli, respectively. The stimulus selection was based on previous studies in which spider and butterfly images were chosen as fear-relevant and fear-irrelevant stimuli^7,43^. While we could have chosen inanimate images, such as toy cars^44^, as neutral stimuli, which are even less likely to evoke fear, we deliberately chose stimuli within the same taxonomic category (i.e., arthropods) to minimize potential visual and perceptual confounds between animate and inanimate objects. Importantly, de Haan et al. (2016)^7^ reported a substantially lower mean score for butterfly fear (3.5 ± 4.0) than for spider fear (21.6 ± 17.7), suggesting that butterflies are generally perceived as less threatening than spiders. For Experiment 1, we selected two spiders and two butterflies as stimuli (Supplementary Fig. 1A). In Experiment 2, we added three additional spider stimuli, resulting in five spiders and two butterflies (Supplementary Fig. 1B). The animations remained in a fixed position, without showing attacking or threatening behavior (spiders walking in place and butterflies flapping their wings; see also Supplementary Videos). These animations were sourced from a 3D model collection site, TurboSquid (https://www.turbosquid.com/), and edited using 3ds Max 2022 (Autodesk). To simulate human binocular vision accurately, we positioned physical cameras slightly to the right and left of the central axis on a 3D editing board. An animation model, centrally located on the editing board, underwent frame-by-frame rendering from the perspectives of both the right and left cameras. We generated a total of 90 rendered frames to cover the 3-s stimulus period (i.e., 30 frames per second). Using Final Cut Pro X 10.4.7, we overlaid these rendered images onto the corresponding footage from the left and right action cameras. As shown in the Supplementary Videos, the spider and butterfly animations were designed to be larger than their real-world size to ensure that participants could see them clearly in the HMDs. Furthermore, in a stereoscopic video environment presented in HMDs, stimuli may appear smaller than in real life; this raises concerns that using realistically life-sized stimuli could attenuate participants’ fear responses, thereby reducing the contrast between fear-relevant and fear-neutral stimuli.

### Experiment 1

#### Participants

We recruited 42 participants (32 females, 10 males; mean age = 28.9 y, range: 19–50) for Experiment 1. All participants were free from neurological or psychiatric conditions and neurodevelopmental disorders and were not using medications that could affect cognitive functions. The experiment was approved by the Swedish Ethical Review Authority, and the experimental procedure was in accordance with the latest version of the approved guidelines. All participants provided written Informed consent. Two females withdrew due to extreme fear of the spider animations used in the main task in a preliminary assessment (see below), resulting in a final sample of 40 participants (30 females, 10 males; mean age = 28.8 y, range: 19–50). We conducted an a priori power analysis using G*Power (version 3.1.9.7)^45^ to determine the appropriate sample size for a paired *t* test comparing subjective fear ratings between visuotactile congruency conditions. The analysis indicated that 41 participants would be required to detect a medium effect size (*dz*) of 0.45 with a power of 0.80 and an alpha level (*α*) of 0.05. We chose a medium effect size due to the lack of specific expectations regarding effect sizes in this experiment.

#### Procedure

At the beginning of the experiment, the participants completed the Fear of Spiders Questionnaire (FSQ), an 18-item self-report measure assessing spider phobia^46^. FSQ scores range from 18 to 126, indicating no fear to high fear, respectively. The mean FSQ score across 42 participants was 47.9 ± 22.1 (mean ± SD; range, 19–103). Individuals scoring 80 or higher on the FSQ were classified as spider sensitive and underwent additional assessment with brief exposure to spider stimuli presented via an HMD. Consequently, two female participants declined to participate in the subsequent experiment. All the participants were explicitly informed of their right to withdraw from the study at any point if they experienced excessive fear in response to the stimuli.

**Fig. 1:**
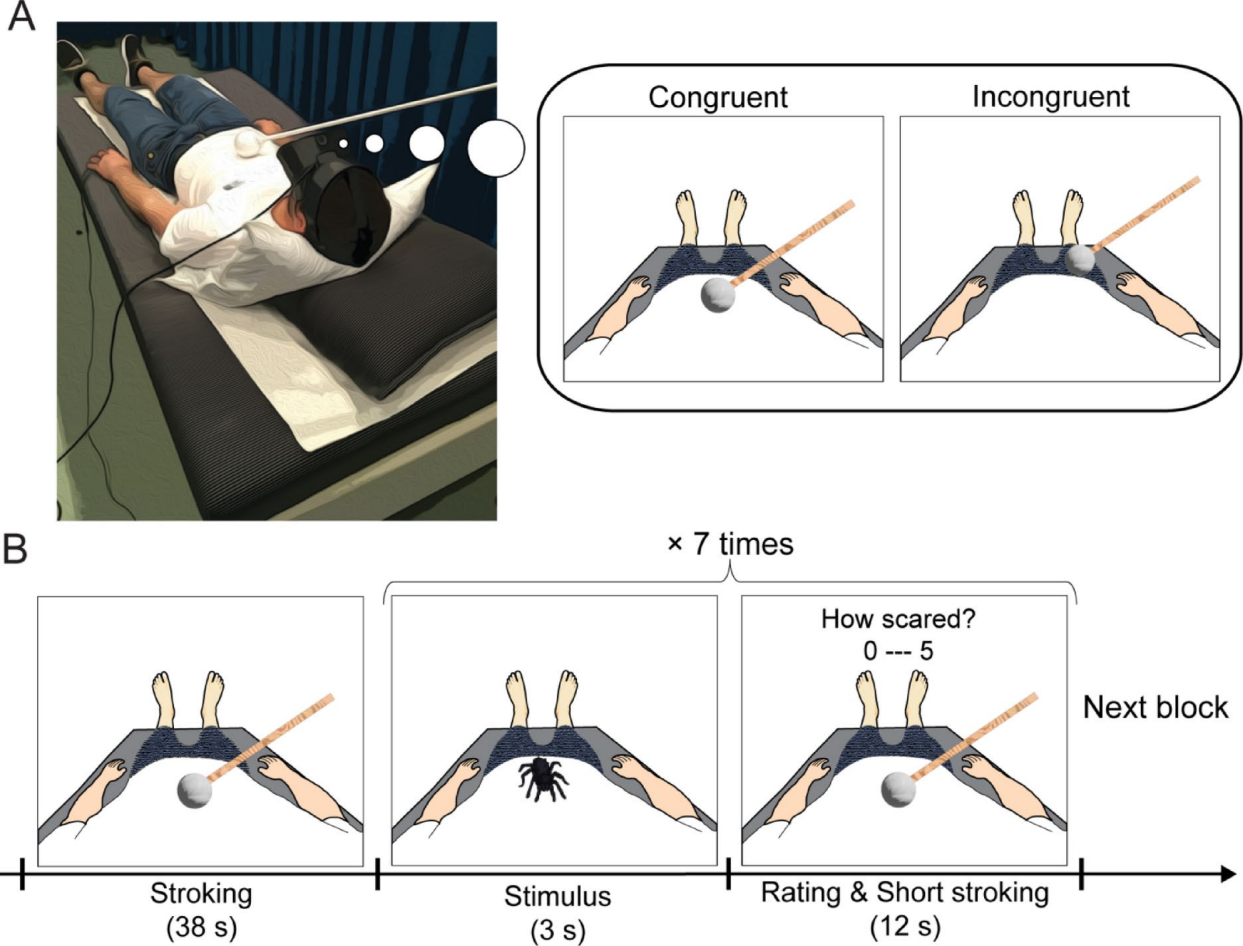
Schematic of the experimental setup and fear-rating task procedure. (**A**) Experimental setup for the full-body illusion paradigm. The participants reclined on a bed while wearing head-mounted displays (HMDs). They viewed videos filming a mannequin’s body parts being stroked with a Styrofoam ball affixed to a wooden rod from the first-person perspective. The participants received physical touches that were either congruent or incongruent with the visual touches applied to the mannequin in terms of timing and location. (**B**) Task timeline. Each block began with a 38-s stroking period. A 3D animation of either a spider or butterfly was subsequently displayed above the mannequin’s abdomen (stimulus period). The participants then reported their fear rating within 4 s while receiving 12 s of visuotactile stimulation (a rating and short stroking period). Each run consisted of two blocks, one with congruent stimulation patterns and one with incongruent visuotactile stimulation patterns. See Supplementary Fig. 1 for spider and butterfly animations and Supplementary Videos 1, 2, and 3 for a trial timeline.

Experiment 1 consisted of two tasks: fear rating and illusion assessment. During the fear rating task, participants lay on a bed fitted with an HMD. Pillows supported their necks, tilting their heads forward (Fig. 1A). The participants were instructed to align their physical bodies with the first-person view of the mannequin’s body shown in the video feed. The SCR was measured during the task, with two skin resistance transducers attached to the index and middle fingers of the participants’ left hand. Each experimental run began with a 38-s stroking period (Fig. 1B). The participants observed the mannequin’s body being stroked with a Styrofoam ball while receiving identical physical strokes from the experimenter. Nine strokes were administered to three body parts, each of which was repeated three times during this period. Individual strokes lasted approximately 1 s, with a 3-s interval between subsequent strokes. One of four predetermined sequence patterns was randomly assigned to each block: sequence 1, A-R-L-R-L-A-R-A-L; sequence 2, L-A-R-R-L-A-L-A-R; sequence 3, R-L-A-L-A-R-A-R-L; and sequence 4, R-A-L-L-R-A-L-A-R (A = abdomen; R = right upper leg; L = left upper leg). During the pre-stimulus and post-stimulus periods, only the mannequin’s body was shown. The durations of these periods were randomized from 4 to 6 s, ensuring a combined time of 10 s. In the 3-s stimulus period, a 3D animation of either a spider or butterfly appeared above the mannequin’s abdomen. Importantly, the animation of a spider or butterfly was presented as a purely visual stimulus, i.e., without any tactile stimulation and independent of the visuotactile stroking. The animations were positioned at either a proximal or distal vertical location (Supplementary Videos 1, 2, and 3). Despite potential differences in the real-world viewing angles of the animations (e.g., more of the lower body would be visible when the animation was distal), identical stimuli were used at both locations to isolate the effect of stimulus location from the confounding influence of the visual angle. During the 12-s rating and short stroking period, participants verbally rated their fear on a six-point Likert scale based on how scared they felt when an animation was presented: zero, no fear; one, very low fear; two, low fear; three, moderate fear; four, high fear; and five, very high fear. Over the course of this period, six strokes were applied visually to the mannequin and physically to the participant’s body. Each stroke lasted 1 s, with a 2-s interval between strokes. The sequence of physical strokes was consistent for each condition: A-R-L-A-R-L for the congruent condition and L-A-R-L-A-R for the incongruent condition (for details in the visuotactile congruency conditions, see below). The sequence of visual strokes was identical in both the congruent and incongruent conditions: A-R-L-A-R-L. The text “How scared” was displayed on the screen for the first four seconds of the period. Although there was no time limit for rating, participants were required to respond promptly once the text was displayed.

We manipulated the temporal and spatial correspondence of strokes visually applied to the mannequin and those applied physically to the participants to alter their sense of the bodily self^19,23^. Under the congruent condition, synchronous strokes were delivered to participants’ own body parts and the corresponding parts of the mannequin. This congruent visuotactile stimulation triggered the illusory sensation that the mannequin was their own body by facilitating multisensory integration of visual information (strokes seen on the mannequin) and somatosensory information (strokes felt on the participant’s body). In contrast, under the incongruent condition, asynchronous strokes were administered to participants’ body parts that did not correspond to the mannequin’s body parts being stroked in the video. For example, participants received a physical touch on their abdomen 1 s after a visual stroke was applied to the right upper leg of the mannequin. This incongruent condition served as a control, maintaining equivalent sensory inputs while altering only the congruency between visual and tactile stimuli.

A sequence of pre-stimulus, stimulus, post-stimulus, and rating and short stroking periods constitutes one trial. Each block consisted of a stroking period followed by seven trials. One run included two blocks (i.e., 14 trials in total), one congruent and one incongruent, in randomized order. The participants completed four runs of the fear rating task. For data analysis, we eliminated the initial trial of each block because its prominence could have a strong impact on the summary of the collected data^47^. Consequently, the total number of analyzed trials was 48 (12 trials per run across four runs).

Following the fear rating task, the participants performed an illusion assessment task. They were positioned in the same posture as in the fear rating task, wearing the HMD. Each run consisted of a stroking period followed by four trials of the fear rating task. Two different types of spider and butterfly animations were presented at two different locations in a random order. Upon completion of the run, the participants answered a questionnaire consisting of four statements about perceptual experiences associated with the full-body illusion and five control statements (Table 1; adopted from previous studies^23,34,48,49^). They rated their agreement with each statement on a 7-point Likert scale ranging from − 3 (strongly disagree) to 3 (strongly agree). The illusion statements involved body ownership (I1) and referral of touch (I2, I3), which are considered the core elements of the multisensory full-body illusion^19,50,51^. The I4 statement (“I felt that the spiders and butterflies I saw were directly above my body.“) was added to explore potential differences in egocentric space perception between the visuotactile conditions. The control statements (C1–C5) assessed the potential effects of suggestibility or task compliance. The order of the statements was randomized among the nine statements. The participants went through two runs, each corresponding to the congruent or incongruent condition. The order of conditions was counterbalanced across participants.

**Table 1:** Questionnaire statements for assessing full-body illusion.

	Item	Statement
Illusion	I1	I felt as if the mannequin’s body was my own body.
	I2	I felt that the touch I experienced was caused by the ball I saw.
	I3	I felt as if the touch of the ball on the mannequin’s body was applied to my body.
	I4	I felt that the spiders and butterflies I saw were directly above my body.
Control	C1	I felt as if I had two bodies at the same time.
	C2	I could no longer feel my body.
	C3	It felt as if I were floating around in the ceiling of the room.
	C4	I felt as if I were located in two places at the same time.
	C5	When I saw the ball moving, I experienced the touch on my back.

#### Skin conductance response

We measured the SCR using the BIOPAC MP160 system (BIOPAC Systems, Inc., Goleta, CA, USA), with two skin resistance transducers attached to the index and middle fingers of the participants’ left hand. Raw signals were recorded at a 2000-Hz sampling rate and high-pass filtered in hardware with a cutoff frequency of 0.5 Hz. We selected this hardware filtering to effectively suppress slow baseline drifts and motion artifacts, ensuring stable detection of phasic SCR responses. The raw signals were downsampled to 100 Hz and low-pass filtered offline with a cutoff frequency of 1 Hz to remove high-frequency noise. We quantified the phasic SCR in response to stimulus presentation as the base-to-peak rise within a window of 0.5–5 s, accounting for the slow rise in the SCR^52–55^. SCR values below 0.02 microsiemens were scored as zero. The phasic SCR was normalized via square-root transformation to correct for its skewed distribution (normalization). Then, within-subject *z* scores were calculated to account for individual differences in SCR amplitudes and enable comparisons across participants (standardization). The preprocessing steps—including low-pass filtering, base-to-peak response detection, square-root normalization, and *z* score standardization—were performed using MATLAB (version R2019b, The MathWorks, Inc., Natick, MA, USA).

### Experiment 2

#### Participants

Forty-one volunteers (21 females, 20 males; mean age = 29.4 y, range: 19–47) participated in Experiment 2. The experiment was approved by the Swedish Ethical Review Authority, and the experimental procedure was in accordance with the latest version of the approved guidelines. All participants provided written Informed consent. We based our sample size calculation on the effect size observed in Experiment 1, specifically when we compared the differences in fear ratings between visuotactile congruency conditions. An a priori power analysis using G*Power suggested a sample size of 41 to detect an effect size (*dz*) of 0.454 with a power of 0.80 and an α of 0.05. Here, we excluded data from three participants who failed to show a significant linear increase in their estimated positions of stimuli from nearest to farthest in a location estimation task (for details, see Supplementary Text: 1. Location estimation task). Thus, the final sample consisted of 38 participants (21 females, 17 males; mean age = 29.2 y, range: 19–47).

#### Procedure

As in Experiment 1, the participants first completed the FSQ to assess their spider phobia. The mean FSQ score across the 41 participants was 37.2 ± 21.9 (mean ± SD; range, 18–104). Despite four participants scoring above 80, all the participants decided to proceed with the subsequent experiment.

Experiment 2 consisted of three tasks: fear rating, location estimation, and illusion assessment tasks. The fear rating task followed a procedure similar to that of Experiment 1, with modifications to the stimulus presentation. During a 3-s stimulus period, a 3D animation of either a spider or a butterfly appeared at one of five vertical positions above the mannequin’s abdomen: closest, second closest, middle, second farthest, or farthest. Here, SCR was not recorded to prioritize the investigation of the subjective experience of fear. Therefore, a post-stimulus period to account for the slow rise in the SCR was not included. The duration of the pre-stimulus period was randomized from 2 to 4 s, ensuring a total time of 21 s for one run (14 trials). One trial constituted a sequence of the pre-stimulus, stimulus, and rating and the short stroking period. Each block consisted of a stroking period followed by seven trials. One run included two blocks (i.e., 14 trials), one congruent and one incongruent, with the order randomized. The participants completed five runs of the fear rating task. Due to time constraints and the absence of SCR recordings during the task, we included the initial trials of each block for the analysis in Experiment 2. The total number of trials was 70 (14 trials per run, across five runs).

After completing the fear rating and location estimation tasks, the participants conducted an illusion assessment task, following the same procedure as in Experiment 1. Here, we omitted the I4 statement (“I felt that the spiders and butterflies I saw were directly above my body.“) due to its ambiguity in quantifying the bodily illusion (for details, see Results). Consequently, the participants rated three statements related to bodily self-consciousness (I1–I3) and five control statements (C1–C5).

### Data analysis

Statistical analyses were performed using RStudio (version 2023.06.1 + 524), an integrated development environment for R (R Core Team, 2023). Normality assessment of questionnaire statement scores, fear ratings, and SCRs was conducted via Shapiro-Wilk tests.

Illusion questionnaire: Following the analysis of previous full-body illusion studies^34,48,49,56^, we calculated illusion scores corrected for suggestibility and response bias by subtracting the mean scores of control statements (C1–C5) from the mean scores of illusion statements (I1–I4 in Experiment 1; I1–I3 in Experiment 2). We assessed the statistical differences in illusion scores between visuotactile congruency conditions via two-tailed paired *t* tests. We also examined the differences in individual statement scores via a two-tailed paired Wilcoxon signed-rank test, as the normality assumption for each statement’s score was violated. Multiple comparisons were corrected by Bonferroni correction.

*Fear rating*: We applied a robust linear mixed-effects model to fear ratings using the rlmer function from the “robustlmm” package^57^. This approach was chosen due to the violation of normality in rating scores for both Experiments 1 and 2, which could increase the likelihood of outliers and skewness in the dataset. In Experiment 1, we modeled fear ratings with visuotactile congruency (1: congruent, -1: incongruent) and stimulus location (1: proximal, -1: distal) as fixed effects and participants as a random effect on the intercept of the fixed effects. As planned follow-up comparisons, we compared fear ratings between stimulus locations (proximal vs. distal) under both congruent and incongruent conditions and between visuotactile congruency conditions (congruent vs. incongruent) under both proximal and distal conditions. The follow-up comparisons were conducted via a two-tailed paired Wilcoxon signed-rank test with Bonferroni correction for multiple comparisons. Furthermore, we compared the proximal-distal differences between visuotactile congruency conditions via a two-tailed paired Wilcoxon signed-rank test to examine how the sense of bodily self modulated fear sensitivity in terms of stimulus proximity. We applied this series of statistical tests separately to fear ratings for spider and butterfly stimuli. In this study, butterfly stimuli served as a control to account for unspecific cognitive responses unrelated to fear. Because incorporating both fear-relevant and neutral stimuli into a single model would complicate the identification of threat-induced effects, we adopted this separate analysis approach.

We also applied a robust linear mixed-effects model to fear ratings in Experiment 2. The model incorporated visuotactile congruency (1: congruent, -1: incongruent) and stimulus location (-2: farthest, -1: second farthest, 0: middle, 1: second closest, and 2: closest) as fixed effects and participants as a random effect on the intercept of the fixed effects. Given that five different spider stimuli were used in the fear rating task of Experiment 2, we incorporated stimulus type (1–5) as a random factor. We only investigated fear ratings for spider stimuli in Experiment 2, as an insufficient number of butterfly trials were collected to permit statistical analysis due to time constraints on the experiment as a whole.

We examined the relationship between the strength of the full-body illusion and subjective fear by combining data from the two experiments (*N* = 78). Considering the variability in fear levels across participants, we first normalized each participant’s fear ratings to calculate *z* scores and then averaged them across all stimulus locations. We then calculated Spearman’s rank-order correlation between the congruent-incongruent difference in illusion scores and the *z* scored fear ratings. We chose Spearman’s correlation due to the non-normal distribution of the data.

*Skin conductance response*: As with the analysis for fear ratings, we applied a robust linear mixed-effects model using the rlmer function from the “robustlmm” package^57^. We modeled SCRs with visuotactile congruency (1: congruent, -1: incongruent) and stimulus location (1: proximal, -1: distal) as fixed effects and participants as a random effect on the intercept of the fixed effects. As planned follow-up comparisons, we compared SCRs between stimulus locations (proximal vs. distal) under both congruent and incongruent conditions and between visuotactile congruency conditions (congruent vs. incongruent) under both proximal and distal conditions. The follow-up comparisons were conducted via a two-tailed paired Wilcoxon signed-rank test with Bonferroni correction for multiple comparisons. We applied this series of statistical tests separately to SCRs evoked by spider and butterfly stimuli.

## Results

### Experiment 1 – Subjective and physiological fear in response to proximal and distal spider and butterfly stimuli relative to the bodily self

#### Illusion questionnaire

**Fig. 2:**
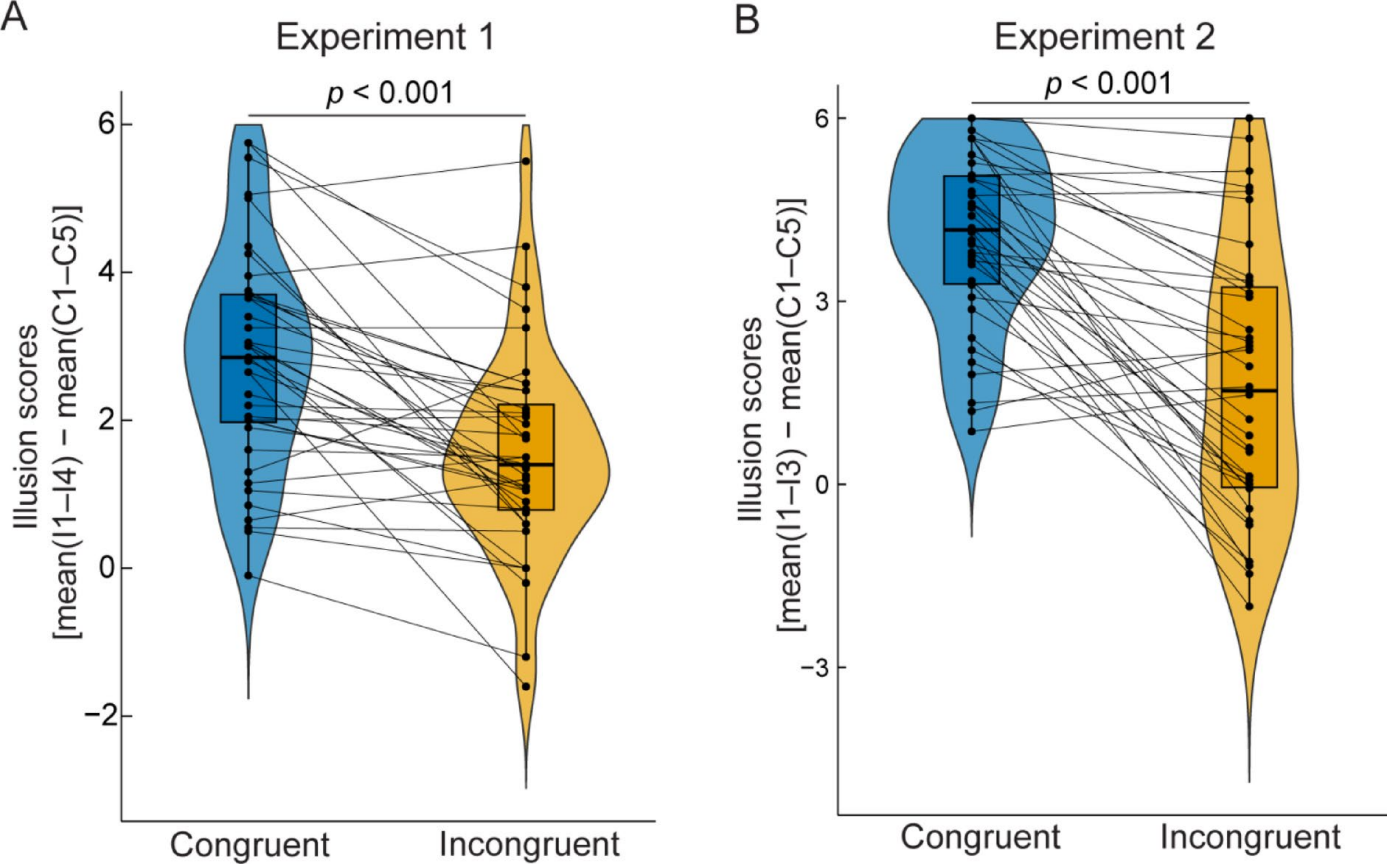
Illusion assessment task results. (**A**) Illusion rating scores in Experiment 1. (**B**) Illusion rating scores in Experiment 2. The scores were calculated by subtracting the mean of the control statement ratings from the mean of the illusion statement ratings. In each box plot, the central horizontal line represents the median, whereas the bottom and top edges of the box indicate the 25th and 75th percentiles, respectively. The whiskers of each box plot extend 1.5 times the interquartile range from each hinge. Each dot corresponds to an individual participant’s data. The rating scores for individual statements in Experiments 1 and 2 are shown in Supplementary Figs. 2 and 4, respectively.

First, we examined the induction of illusory body ownership over the mannequin by congruent visuotactile stimulation. The illusion scores under the congruent condition (*M* = 2.82, *SD* = 1.47) were significantly higher than those under the incongruent condition (*M* = 1.54, *SD* = 1.36; *t*(39) = 5.69, *p* < 0.001, Cohen’s *d* = 0.90, two-tailed paired *t* test; Fig. 2A). All illusion statement scores, except for the I4 statement, were significantly higher under the congruent condition than under the incongruent condition (I1: *Z* = 3.22, *p* = 0.001, rank-biserial correlation (*ρ*_rb_) = 0.71; I2: *Z* = 3.68, *p* < 0.001, *ρ*_rb_ = 0.75; I3: *Z* = 4.56, *p* < 0.001, *ρ*_rb_ = 0.95; I4: *Z* = 2.29, *p* = 0.019, *ρ*_rb_ = 0.61, two-tailed paired Wilcoxon signed-rank test with a Bonferroni-corrected alpha level of 0.05/4 = 0.0125; Supplementary Fig. 2A). Under the congruent condition, the means and medians for the I1, I2, and I3 statements were positive, suggesting that the majority of participants affirmed feeling illusory body ownership over the mannequin’s body and sensing the touches applied to it. In contrast, we found no significant difference in the ratings of the control statements between the congruent and incongruent conditions (C1: *Z* = -1.30, *p* = 0.19, *ρ*_rb_ = -0.33; C2: *Z* = 0.14, *p* = 0.90, *ρ*_rb_ = 0.037; C3: *Z* = -0.20, *p* = 0.85, *ρ*_rb_ = -0.058; C4: *Z* = -0.28, *p* = 0.79, *ρ*_rb_ = -0.069; C5: *Z* = 0.36, *p* = 0.75, *ρ*_rb_ = 0.13; Supplementary Fig. 2B). The means and medians for these control statements were negative under both conditions. Collectively, these results indicate that congruent visuotactile stimulation induced a significantly illusory sensation of owning the mannequin’s body, which was not observed with incongruent stimulation, suggesting that the two types of visuotactile stimulation served as effective experimental manipulations of participants’ sense of bodily self.

We incorporated established illusion statements (I1, I2, and I3) from previous studies^23,34,37,48,49^ while newly introducing the I4 statement to examine potential changes in participants’ egocentric perceptions of visual stimuli. However, in retrospect, this new statement might have been confusing and insufficiently clear, as it required participants to simultaneously assess two aspects of the illusory experience: the perception of stimuli located above their bodies and the feeling of ownership over the mannequin’s body. This ambiguity might have resulted in the inconsistency between the scores of the I4 statement and the other illusion statements. Given these challenges, we concluded that the I4 statement was ineffective for accurately quantifying the bodily illusion and excluded it from the questionnaire statements in Experiment 2.

#### Fear rating

**Fig. 3:**
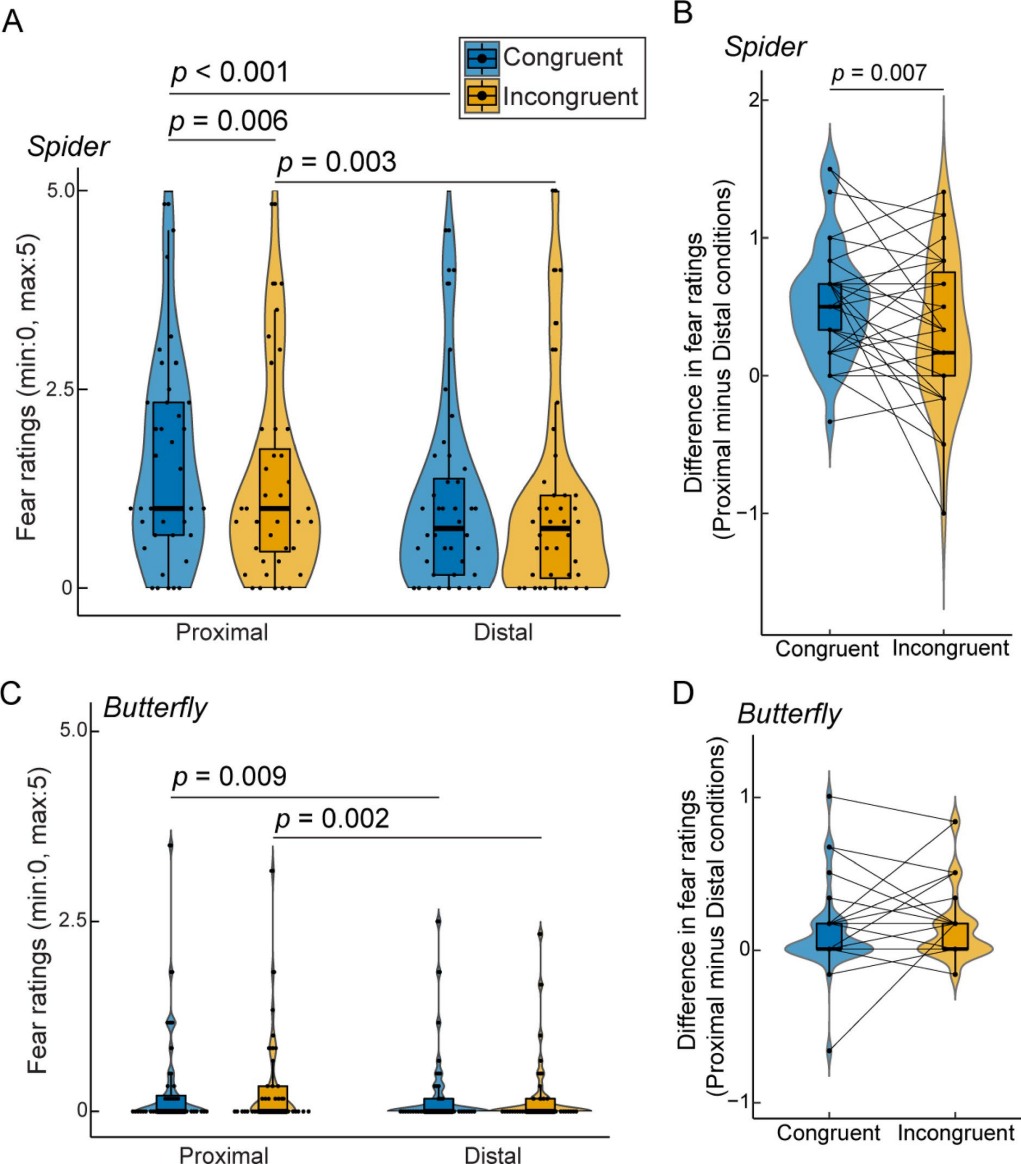
Fear rating results in Experiment 1. (**A**) Fear ratings for fearful spider stimuli across visuotactile congruency and stimulus location conditions. (**B**) Difference in fear ratings between proximal and distal spider stimuli under each visuotactile congruency condition. (**C**) Fear ratings for neutral butterfly stimuli across visuotactile congruency and stimulus location conditions. (**D**) Difference in fear ratings between proximal and distal butterfly stimuli under each visuotactile congruency condition. In each box plot inside the violin plots, the central horizontal line indicates the median, and the bottom and top edges of the box correspond to the 25th and 75th percentiles, respectively. The whiskers of each box plot extend 1.5 times the interquartile range from each hinge. Each dot represents one participant.

Next, we examined the effect of the sense of bodily self on subjective fear. We first analyzed the fear ratings for the spider stimuli (Fig. 3A). A robust linear mixed-effects model revealed a significant main effect of both visuotactile congruency (*β* = 0.062, *SE* = 0.024, *t*_117_ = 2.59, *p* = 0.011) and stimulus location (*β* = 0.17, *SE* = 0.024, *t*_117_ = 7.06, *p* < 0.001). The interaction between the two factors was also significant (*β* = 0.049, *SE* = 0.024, *t*_117_ = 2.04, *p* = 0.044). Follow-up planned comparisons revealed that the fear ratings for proximal spiders were significantly higher under the congruent condition than under the incongruent condition (*Z* = 2.75, *p* = 0.006, *ρ*_rb_ = 0.57, two-tailed Wilcoxon signed-rank test with a Bonferroni-corrected alpha-level of 0.05/4 = 0.0125). However, no significant difference was found in the fear ratings for distal spiders between the two visuotactile-congruency conditions (*Z* = 0.37, *p* = 0.73, *ρ*_rb_ = 0.087). As expected, the fear ratings for the proximal spider were significantly higher than those for the distal spider under both congruent (*Z* = 4.78, *p* < 0.001, *ρ*_rb_ = 0.97) and incongruent conditions (*Z* = 3.00, *p* = 0.003, *ρ*_rb_ = 0.62). Furthermore, the difference in the fear ratings between the proximal and distal spider stimuli was significantly greater under the congruent condition than under the incongruent condition (*Z* = 2.69, *p* = 0.007, *ρ*_rb_ = 0.54; Fig. 3B). These findings suggest that subjective fear is sensitively modulated by stimulus location during the body ownership illusion.

With respect to the fear ratings for the butterfly stimuli (Fig. 3C), a robust linear mixed-effects model revealed a significant main effect of stimulus location (*β* = 0.029, *SE* = 0.0067, *t*_117_ = 4.29, *p* < 0.001). However, neither the main effect of visuotactile congruency (*β* = -0.0024, *SE* = 0.0067, *t*_117_ = -0.37, *p* = 0.72) nor the interaction (*β* = -0.0027, *SE* = 0.0067, *t*_117_ = -0.40, *p* = 0.69) reached statistical significance. Planned comparisons, consistent with the linear mixed-effects model results, revealed significantly higher fear ratings for proximal than for distal butterfly stimuli under both congruent (*Z* = 2.61, *p* = 0.009, *ρ*_rb_ = 0.77, two-tailed Wilcoxon signed-rank test with a Bonferroni-corrected alpha-level of 0.05/4 = 0.0125) and incongruent conditions (*Z* = 3.11, *p* = 0.002, *ρ*_rb_ = 0.84). In contrast, no significant difference was found between the congruent and incongruent conditions for either proximal (*Z* = 0.33, *p* = 0.76, *ρ*_rb_ = 0.088) or distal stimuli (*Z* = 0.66, *p* = 0.53, *ρ*_rb_ = 0.21). Additionally, the proximal-distal difference in fear ratings did not significantly differ between the visuotactile congruency conditions (*Z* = -0.61, *p* = 0.55, *ρ*_rb_ = -0.16; Fig. 3D). Thus, the control findings eliminate the possibility that mere saliency or surprise effects amplified by stimulus appearance were responsible for the greater sensitivity of subjective fear to stimulus location under the congruent condition. Rather, the above findings indicate that bodily self-perception interacts with the proximity of fear-relevant stimuli in modulating defensive fear responses.

#### Skin conductance response

**Fig. 4:**
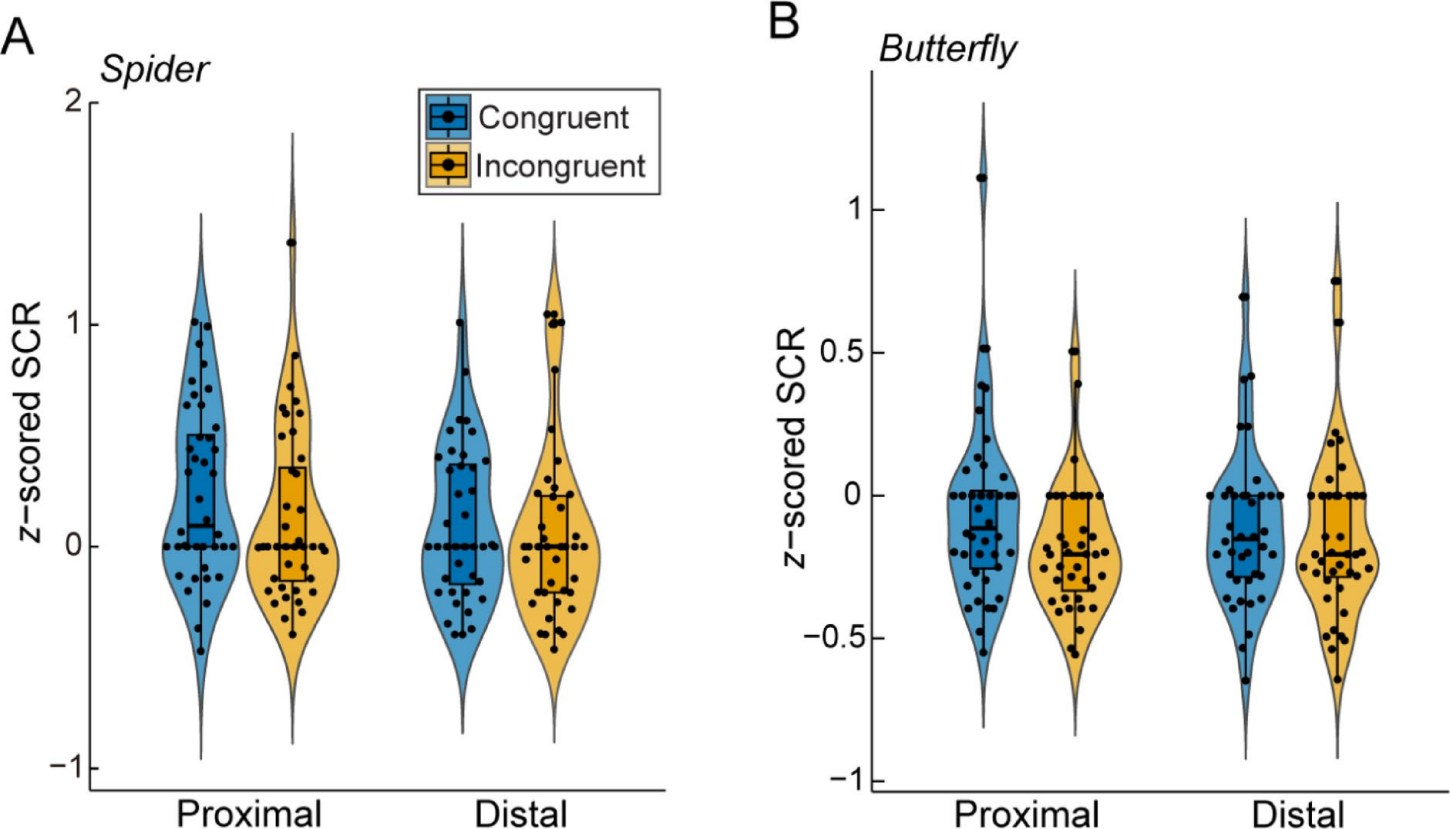
Skin conductance responses to spider and butterfly stimuli in Experiment 1. (**A**) Mean SCRs to proximal and distal spider stimuli under the congruent and incongruent visuotactile congruency conditions. (**B**) Mean SCRs to proximal and distal butterfly stimuli under the congruent and incongruent visuotactile congruency conditions. In each box plot within the violin plots, the central horizontal line indicates the median, and the bottom and top edges of the box correspond to the 25th and 75th percentiles, respectively. The whiskers of each box plot extend 1.5 times the interquartile range from each hinge. Each dot indicates one participant.

To assess physiological responses to spider stimuli, we analyzed SCRs using a robust linear mixed-effects model (Fig. 4A). This model incorporated visuotactile congruency and stimulus location as fixed effects and participants as a random effect on the intercept of the fixed effects. No significant main effects were observed for visuotactile congruency (*β* = 0.053, *SE* = 0.030, *t*_156_ = 1.77, *p* = 0.079) or stimulus location (*β* = 0.056, *SE* = 0.030, *t*_156_ = 1.87, *p* = 0.063), nor was a significant interaction found between these factors (*β* = 0.013, *SE* = 0.030, *t*_156_ = 0.43, *p* = 0.67). Post hoc comparisons revealed no significant differences in SCRs between the congruent and incongruent conditions for either proximal (*Z* = 1.21, *p* = 0.23, *ρ*_rb_ = 0.26) or distal spiders (*Z* = 0.69, *p* = 0.50, *ρ*_rb_ = 0.15, two-tailed Wilcoxon signed-rank test with a Bonferroni-corrected alpha-level of 0.05/4 = 0.0125). Additionally, we found no significant differences between proximal and distal locations under either congruent (*Z* = 1.51, *p* = 0.13, *ρ*_rb_ = 0.32) or incongruent conditions (*Z* = 0.78, *p* = 0.45, *ρ*_rb_ = 0.17). Next, we performed a time series analysis using a 100-ms sliding time window with 10-ms shifts. The square-root-transformed signal averaged within each time window under the congruent-proximal condition was compared with that under the other three conditions (i.e., the congruent-distal, incongruent-proximal, and incongruent-distal conditions). As shown in Supplementary Fig. 3A, the highest peak was found under the congruent-proximal condition. However, the statistical analysis did not reveal any significant differences (*p* < 0.01, uncorrected), likely due to the large interindividual variance in the SCR.

We also analyzed SCRs in response to butterfly stimuli (Fig. 4B). A robust linear mixed-effects model revealed no significant main effect of visuotactile congruency (*β* = 0.033, *SE* = 0.019, *t*_156_ = 1.72, *p* = 0.087), no significant main effect of stimulus location (*β* = 0.0025, *SE* = 0.019, *t*_156_ = 0.13, *p* = 0.90), and no significant interaction (*β* = 0.019, *SE* = 0.019, *t*_156_ = 0.98, *p* = 0.33). Post hoc planned comparisons revealed no significant differences between visuotactile congruency conditions for either proximal (*Z* = 1.89, *p* = 0.061, *ρ*_rb_ = 0.51) or distal stimuli (*Z* = 0.88, *p* = 0.39, *ρ*_rb_ = 0.21, two-tailed Wilcoxon signed-rank test with a Bonferroni-corrected alpha-level of 0.05/4 = 0.0125). Similarly, no significant differences were found between the stimulus location conditions under either the congruent (*Z* = 0.99, *p* = 0.33, *ρ*_rb_ = 0.24) or incongruent condition (*Z* = -0.68, *p* = 0.51, *ρ*_rb_ = -0.18). The peak responses were observed approximately 3 s after stimulus onset in the time course of the SCR signal (Supplementary Fig. 3B). However, a time series analysis revealed no significant differences among the four conditions (*p* < 0.01, uncorrected).

### Experiment 2 – Subjective fear in response to spider stimuli at five distances from the bodily self

In Experiment 2, we examined how subjective fear changes as a function of the distance of visual stimuli from the bodily self, presenting stimuli at five different distances from a mannequin: closest, second closest, middle, second farthest, and farthest.

#### Illusion questionnaire

We first assessed whether visuotactile stimulation could successfully induce the full-body ownership illusion. The illusion rating score was significantly higher under the congruent condition (*M* = 4.03, *SD* = 1.42) than under the incongruent condition (*M* = 1.64, *SD* = 2.20; *t*(37) = 6.99, *p* < 0.001, Cohen’s *d* = 1.13, two-tailed *t* test; Fig. 2B). The ratings for individual illusion statements were also significantly higher under the congruent condition than under the incongruent condition (I1: *Z* = 4.49, *p* < 0.001, *ρ*_rb_ = 0.94; I2: *Z* = 4.17, *p* < 0.001, *ρ*_rb_ = 0.89; I3: *Z* = 4.26, *p* < 0.001, *ρ*_rb_ = 0.94, two-tailed Wilcoxon signed-rank test with a Bonferroni-corrected alpha-level of 0.05/3 = 0.0167; Supplementary Fig. 4A). In contrast, the control statements, except for the C1 statement, showed no significant differences between visuotactile congruency conditions (C1: *Z* = -3.00, *p* = 0.003, *ρ*_rb_ = -0.70; C2: *Z* = -0.28, *p* = 0.79, *ρ*_rb_ = -0.069; C3: *Z* = -0.47, *p* = 0.66, *ρ*_rb_ = -0.15; C4: *Z* = -1.49, *p* = 0.13, *ρ*_rb_ = -0.39; C5: *Z* = 0.94, *p* = 0.41, *ρ*_rb_ = 0.47, a Bonferroni-corrected alpha-level of 0.05/5 = 0.01; Supplementary Fig. 4B). While the C1 statement indicated a significant difference between the visuotactile congruency conditions, its ratings were higher under the incongruent condition, in contrast with the pattern observed in the illusion statements. Thus, the above findings support the successful induction of the full-body illusion in Experiment 2.

#### Fear rating

**Fig. 5:**
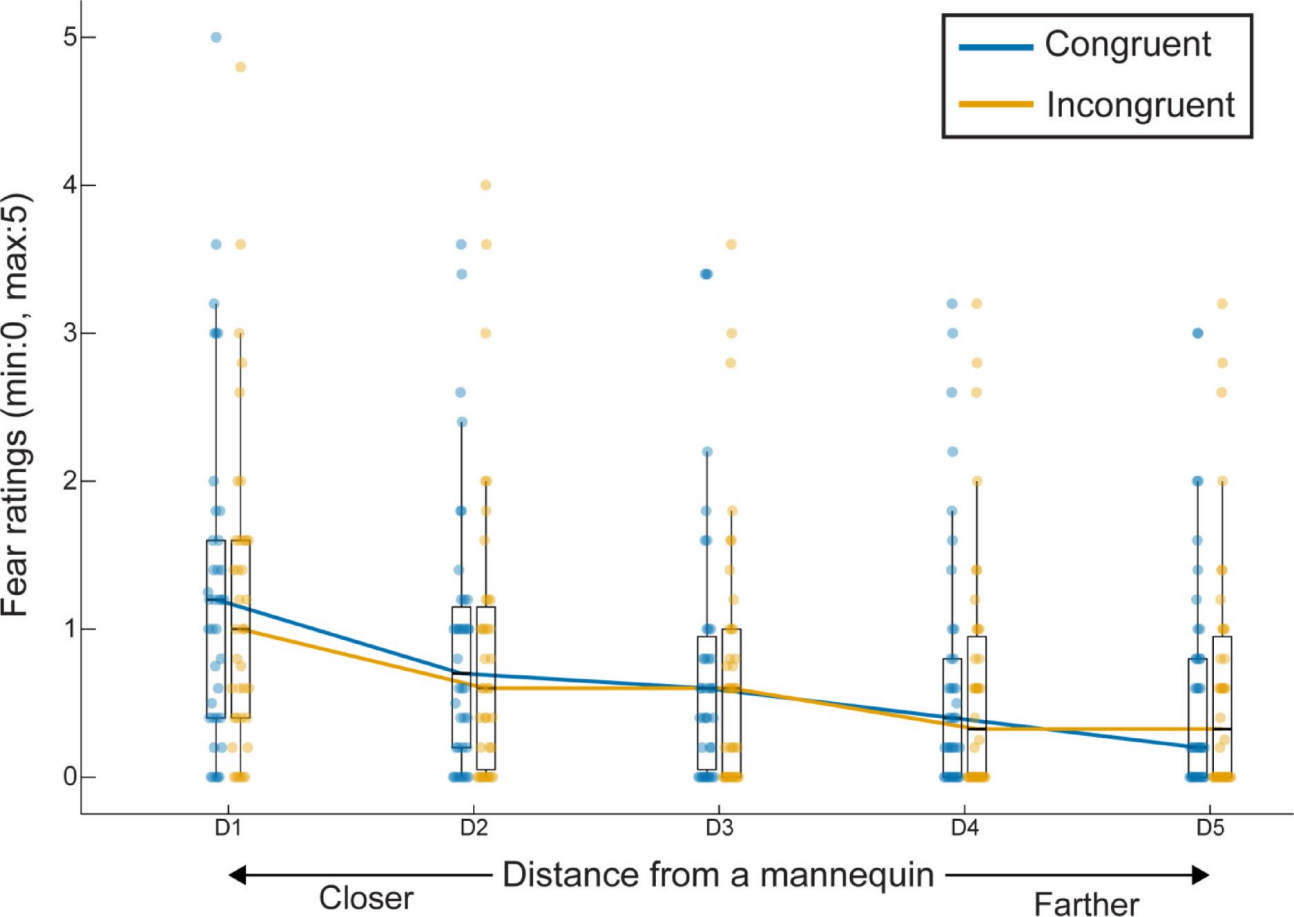
Fear rating result in Experiment 2. Fear ratings elicited by spider stimuli presented at five distances from a mannequin (D1: closest, D2: second closest, D3: middle, D4: second farthest, D5: farthest) under congruent (blue-green) and incongruent (orange) visuotactile congruency conditions. Each box plot shows the median (central horizontal line), 25th and 75th percentiles (bottom and top edges of the box, respectively), and whiskers extending 1.5 times the interquartile range from each hinge. Individual data points represent single participants.

A robust linear mixed-effects model indicated significant main effects for both visuotactile congruency (*β* = 0.023, *SE* = 0.012, *t*_1846_ = 1.98, *p* = 0.048) and stimulus location (*β* = 0.12, *SE* = 0.0082, *t*_1846_ = 15.0, *p* < 0.001; Fig. 5), whereas the interaction between these factors was not significant (*β* = 0.0014, *SE* = 0.0082, *t*_1846_ = 0.17, *p* = 0.86). As a further exploration of the relationship between fear ratings and stimulus location, we fitted a linear regression to each participant’s ratings as a function of stimulus location. The regression slopes were negative under both the congruent (*V* = 13.5, *p* < 0.001, *ρ*_rb_ = -0.95, two-tailed Wilcoxon signed-rank test) and incongruent conditions (*V* = 9.0, *p* < 0.001, *ρ*_rb_ = -0.96). However, these slopes did not differ significantly between visuotactile congruency conditions (*Z* = -0.48, *p* = 0.64, *ρ*_rb_ = -0.10). Thus, these findings demonstrate that increasing the distance of fear-relevant stimuli from the mannequin led to a reduction in participants’ subjective fear; however, visuotactile congruency did not interact significantly with this distance-dependent effect. Although the interaction was not observed, Experiment 2 replicated the main effect that congruent visuotactile stimulation enhances subjective fear.

#### Correlation between subjective fear and full-body illusion strength

**Fig. 6:**
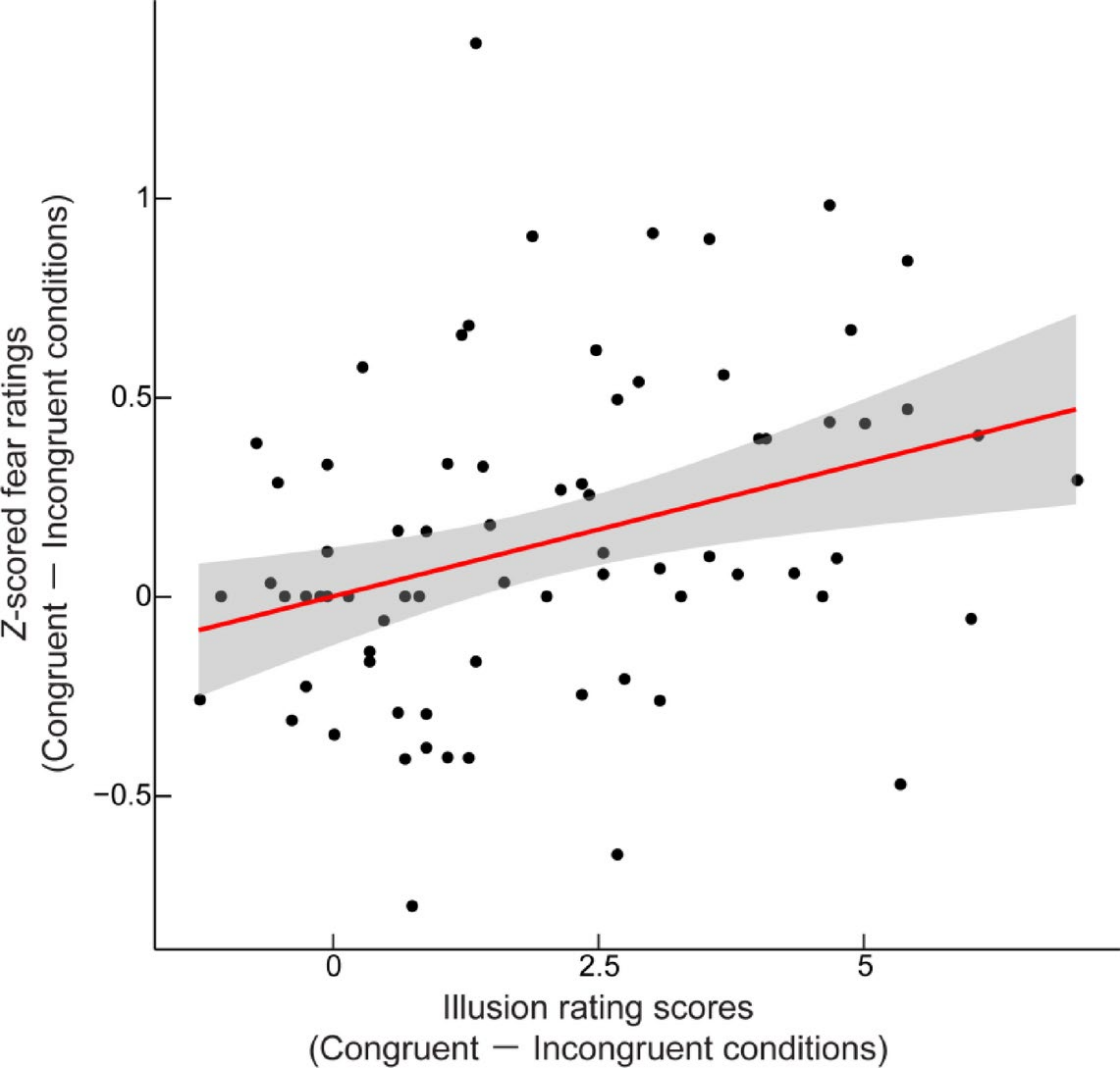
Correlation between illusion strength and subjective fear. The scatter plot depicts the relationship between illusion rating scores and *z* scored fear ratings. Illusion rating scores were calculated as the mean difference between illusion and control statement ratings. Each data point corresponds to an individual participant (*N* = 78). The red line shows the linear regression, with the gray shaded area indicating the 95% confidence interval. A significant positive correlation was observed between illusion rating scores and fear ratings (Spearman’s *ρ* = 0.38, *p* < 0.001).

To investigate the relationship between illusion strength and fear ratings, we calculated Spearman’s rank-order correlation using combined data from both experiments (*N* = 78). We normalized the fear ratings by transforming them into *z* scores and calculating the mean across all stimulus locations. Consequently, a significant positive correlation was found between the differences in illusion ratings and fear ratings between the congruent and incongruent conditions (Spearman’s *ρ* = 0.38, *p* < 0.001, two-sided; Fig. 6). Additionally, we observed significant correlations between the illusion ratings and the fear ratings of spider stimuli at both the closest location (*ρ* = 0.23, *p* = 0.044) and the farthest location (*ρ* = 0.32, *p* = 0.005). The difference between these two correlations did not reach statistical significance (*r* test: *z* = -0.84, *p* = 0.40). These findings suggest that the strength of the sense of bodily self is associated with the level of subjective fear and that this relationship appears to be irrespective of the spatial proximity of the fear-relevant stimuli.

## Discussion

In this study, we sought to characterize the functional role of the sense of bodily self in the interplay between threat proximity and defensive fear. We hypothesized that humans perceive threats as being proximal or distal based on their distance from the perceived location of the bodily self. This finding is supported by the findings of Experiment 1, which revealed that subjective fear ratings were more sensitive to the location of spider stimuli under the congruent condition (Fig. 3B). The congruent visuotactile stimulation provided participants with the perceptual experience that a mannequin’s body was their own (i.e., manipulation of body ownership^23^) and that their bodily self was located in the space occupied by the mannequin displayed on the HMD screen (i.e., manipulation of the sense of self-location^21,33,34,58^). In this situation, the brain remaps the egocentric reference frame, relocating its origin to the perceived self-location, and updates the egocentric spatial representation centered on the mannequin^19,23,34,38^. Consequently, a bodily self-centered spatial relationship to the threats could be established. Conversely, incongruent visuotactile stimulation, which reduces or diminishes the illusory feeling of body ownership over the mannequin, also reduces the spatial feeling of being located in the same place as the mannequin^34^; this could lead the brain to dissociate the external visual world where the threat appeared from the bodily self, making participants no longer ‘feel part’ of the visual world in the embodied sense of the word we theorize. This proposed cognitive mechanism is consistent with previous research demonstrating that full-body illusions shift the spatial representation around the self-body (i.e., the PPS) from the physical body location to the space encompassing a virtual body identified as one’s own^5,36,59^. Furthermore, an fMRI study using the rubber hand illusion revealed that the premotor-parietal network is responsible for encoding visual stimuli in hand-centered spatial coordinates remapped onto a rubber hand^60,61^. Regarding possible neural mechanisms underlying our behavioral findings, this premotor-parietal network could encode threat locations in the egocentric coordinate system centered on the mannequin’s body and interact with fear circuits (e.g., the amygdala, insula, anterior cingulate cortex, midbrain, and brainstem nuclei^12,44,62,63^), reflecting spatial information on defensive fear. Further neuroimaging studies are needed for a deeper understanding of the neurocognitive mechanisms underlying the interplay between the sense of bodily self, spatial perception, and fear processing in the human brain.

In Experiment 2, in which stimuli appeared at one of five locations, we did not find a significant interaction between visuotactile congruency and stimulus location (Fig. 5). This nonsignificant result may be caused by rapid habituation resulting from frequent exposure to spider stimuli, potentially leading to reduced sensitivity in fear ratings across conditions (see also Limitations). Nonetheless, in line with Experiment 1, we identified a main effect of visuotactile congruency. Moreover, we demonstrated a significant positive correlation between fear ratings and illusion ratings (Fig. 6), indicating that the stronger the sense of bodily self was experienced, the more afraid the participants were of the appearance of spider stimuli. Interestingly, this correlation persists regardless of the proximal or distal location of the stimuli. With respect to the threat imminence continuum model^1^, spider stimuli presented at distal locations could be categorized as post-encounter threats rather than pre-encounter or circa-strike threats. In the post-encounter context, threats are believed not yet to be the subject of imminent danger; however, they may elicit anxiety in anticipation of their potential future behaviors^2^. Our findings from Experiment 2 suggest that the bodily illusion enhances the level of threat encounter anxiety as well as the sensitivity to PPS violations. A possible explanation is that the sense of self-location pronounced by the full-body illusion induced a feeling of shared space between the bodily self and fear-relevant stimuli. Such a strong sense of immersion could evoke a realistic sensation of threat encounter, escalating anxiety even though the spiders were at a distance from the mannequin. Likewise, VR studies have reported associations between the sense of immersion and emotional responses in both fear-related and non-fear-related contexts^14,15,18,64^.

We observed no significant effects of visuotactile congruency or stimulus location on SCRs in Experiment 1 (Fig. 4), which was inconsistent with the subjective rating results. Two possible explanations exist for this inconsistency: (1) a dissociation between the neural substrates mediating the subjective and physiological components of fear and (2) rapid habituation of the physiological fear response. Fear studies commonly employ fear ratings and SCRs as subjective and objective measures of participants’ fearful mental states, respectively. Although these measures have often been used interchangeably, recent research casts doubt on the one-to-one mapping between them. Taschereau-Dumouchel et al. (2019) demonstrated that subjective reports and physiological reactivity to threats originated from distinct neural substrates^47,65^. In light of such recent debates, we can reasonably speculate that the sense of bodily self more strongly influences subjective fear experiences than does physiological fear reactivity. Regarding the second explanation, it is commonly observed that the magnitude of SCRs decreases with repeated exposure to threatening stimuli^66^. A full-body illusion study revealed a nonlinear (exponential) trend of SCR habituation when knife threats were repeatedly presented to a virtual body perceived as one’s own^48^. In our study, the spider stimuli may not have necessarily elicited intense fear in the majority of the participants, potentially leading to rapid habituation within the first few trials. Further research is needed to clarify the cause of the discrepancy between subjective and physiological results by, for example, presenting more realistic fear-relevant stimuli, employing different types of physiological measures, or using neuroimaging techniques.

While the current study focuses on the relationship between bodily self-perception and fear responses to the proximity of a fear-relevant stimulus, previous research has also explored the association between the sense of body ownership and emotional reactions triggered by physical threats directed toward body parts (rubber hand illusion^67–72^ and full-body illusion^23,34,48,56,73^). For example, Petkova and Ehrsson (2008)^23^ reported that higher SCRs were detected when participants observed a knife cutting over a mannequin’s belly while experiencing a full-body illusion. Similarly, Fan et al. (2021)^70^ reported elevated threat-evoked SCR when the middle finger of a rubber hand was pricked by a needle attached to a syringe during the rubber hand illusion. Such direct physical threats by sharp objects could cause tissue damage and pain; consequently, participants anticipate pain and experience anxiety when they see the sharp objects approaching the rubber hand or mannequin during the body ownership illusion, similar to—but weaker than—how they would feel if their actual bodies were threatened in the same way. In contrast, our study addresses phylogenetic threats, such as spiders, which have shaped evolutionary pressures that encourage the avoidance of close proximity and evoke subjective fear, even though they do not necessarily cause physical harm^74^. Here, we demonstrated that congruent visuotactile stimulation, which induced an illusory sensation of body ownership over a mannequin, led to greater fear to the proximity of phylogenetic fear-relevant stimuli than did the incongruent control condition (Fig. 3A). This finding suggests that bodily self-perception interacts with evolutionarily conserved fear mechanisms driven by the spatial proximity of phylogenetic threats.

## Limitations

A limitation of this study is that the spider stimuli used were potentially insufficient to elicit a robust fear response. Our choice of 3D spider models as threatening stimuli was based on previous research reporting high fear ratings, strong SCRs, and activations in fear-related brain regions, especially the amygdala, when spider images or videos were presented even to nonphobic participants^44,47,63^. However, although these 3D models appeared realistic when inspected on a 2D computer screen (Supplementary Fig. 1), their perceived authenticity may have been reduced during the process of superimposing them onto recorded videos, possibly due to resolution degradation or other technical constraints. Moreover, the repeated exposure to spider stimuli (28 trials in Experiment 1 and 50 trials in Experiment 2) may have resulted in substantial habituation. Physiological and neural responses are sensitive to stimulus novelty and tend to attenuate with repeated exposures^75,76^. Further studies should investigate the modulation of physiological fear responses by the full-body illusion, enhancing stimulus authenticity and novelty by using various types of phylogenetic stimuli other than spiders with a high-quality display system.

In Experiment 1, the SCR was recorded as a physiological measure of fear. However, the results were negative and inconclusive. Whether other relevant physiological measures could reveal differences in fear responses remains an open possibility that has yet to be tested. Heart rate recordings, for example, could have captured physiological components of fear responses, as previous studies revealed a link between heart rate and fear/threat emotions and fear learning^77,78^. Pupil size might have served as a complementary measure, as exposure to threatening stimuli was shown to induce greater pupil dilation than did exposure to neutral stimuli^79–81^. Thus, our exclusive use of SCR recording is a potential limitation of this study, and future research should consider these additional physiological measures to provide a more comprehensive picture of fear responses.

A commonly used method for assessing PPS boundaries is a multisensory interaction paradigm in which participants respond as quickly as possible to a tactile stimulus presented simultaneously with a visual or auditory stimulus located either near to or far from their body^5,36,82–84^. This paradigm detects response facilitation for near-body stimulation, thereby characterizing PPS boundaries. In the present study, we did not adopt this paradigm, which limits direct comparisons with previous PPS findings—a potential theoretical limitation. Although investigating PPS boundaries could have provided valuable insights into the role of PPS in the spatial coding of fear-relevant stimuli, our primary goal was to examine how a body ownership illusion modulates both subjective and physiological measures of fear in a distance-dependent manner—an intersection between bodily self-consciousness and fear research. Furthermore, practical constraints restricted the feasibility of implementing a PPS multisensory interaction task within the current full-body illusion. Our illusion induction procedure required 38 s, and incorporating a multisensory interaction task would have required a large number of trials to obtain robust results, raising concerns about an excessively prolonged experimental duration and the potential risk of habituation to fear stimuli.

Another possible limitation of our study is the unequal distribution of male and female participants in Experiment 1, which could introduce bias into the results. Although the intensity of fear elicited by phylogenetic threats is generally believed to differ between males and females, we did not find a significant difference in the fear of spider questionnaire scores between sexes (two-tailed *t* test, *t*(38) = 1.15, *p* = 0.26). More importantly, when we added sex (1: male vs. -1: female) as a fixed effect in a robust LMEM for subjective fear ratings of the spider stimuli, we found no significant interactions between visuotactile congruency and sex (*t*_114_ = -1.18, *p* = 0.24), between stimulus location and sex (*t*_114_ = 1.41, *p* = 0.16), or among these three factors (*t*_114_ = -0.32, *p* = 0.75). Consequently, the imbalance in the number of male and female participants was unlikely to have biased our findings regarding the effect of the sense of bodily self on subjective fear.

## Conclusions

In summary, the current study revealed two functional roles of the sense of bodily self in human fear experiences: increasing sensitivity to margin-of-safety violations and increasing levels of fear/anxiety in response to the appearance of phylogenetic threat. These findings advance our basic understanding of how bodily self-perception shapes our emotional experiences and provide informative insight into psychiatric disorders characterized by extreme fear or anxiety, such as phobias and posttraumatic stress disorder. Faul et al. (2020)^16^ demonstrated that fear memories conditioned by proximal threats in immersive 3D environments presented greater resistance to extinction. This finding suggests that threats perceived in proximity to one’s selfhood could exacerbate psychiatric symptoms. A deeper understanding of the link between bodily self-perception and anxiety disorders will lay the groundwork for developing effective treatments for patients experiencing severe fear or anxiety.

## Electronic supplementary material

Below is the link to the electronic supplementary material.

Supplementary Material 1

Supplementary Material 2

Supplementary Material 3

Supplementary Material 4

## Data Availability

All data are available as open data via the Open Science Framework (OSF) repository at https://osf.io/u2xn8/?view_only=9089f9d472e5414a80361bc3876d33f9.

## Code availability

The original codes used to analyze the collected data are available via the OSF repository at https://osf.io/u2xn8/?view_only=9089f9d472e5414a80361bc3876d33f9.
