# Supplementary material for: Bodily illusion enhances subjective fear of safety-margin violations surrounding the bodily self
Source: Sci Rep. 2025 Apr 1;15:11195. doi: 10.1038/s41598-025-95976-x (PMC11961577; doi:10.1038/s41598-025-95976-x)
Supplement: Supplementary file 4 — Supplementary Material 4 [file 41598_2025_95976_MOESM4_ESM.docx]

Supplementary Information

**Bodily illusion enhances subjective fear of safety-margin violations surrounding the bodily self**

Ryu Ohata and H. Henrik Ehrsson

Correspondence to R.O (ryu.oohata@gmail.com)

**Supplementary Texts**

1. Location estimation task

2. Inclusion of initial trials of each block

**Supplementary Figures**

Supplementary Fig. 1. 3D spider and butterfly animations

Supplementary Fig. 2. Rating scores for individual statements in the illusion-assessment task in Experiment 1.

Supplementary Fig. 3. Time courses of SCR in Experiment 1

Supplementary Fig. 4. Rating scores for individual statements in the illusion-assessment task in Experiment 2.

**Captions for Supplementary Videos**

Supplementary Video 1. Example of proximal spider stimulus for experimental participants

Supplementary Video 2. Example of distal spider stimulus for experimental participants

Supplementary Video 3. Example of proximal butterfly stimulus for experimental participants

**Supplementary Texts**

**1. Location estimation task**

Between fear rating and illusion assessment tasks, we conducted a location estimation task in Experiment 2. Participants engaged in this task to assess their perception of interval lengths between five stimulus locations. The task began with the presentation of the mannequin’s body from a first-person perspective on a head-mounted display screen. Following a 1-s ready period, a 3D white sphere appeared near the ceiling of the room for 1 s. After an interval of 1 s, a spider animation was presented above the mannequin’s abdomen for 3 s. Upon the spider stimulus disappearing, the text "Estimate distance, 0 --- 100" was displayed for 5 s. Participants verbally reported their estimated distance from the mannequin's abdomen to the spider stimulus within this 5-s period. They were informed that the white sphere's location represented the reference point of 100, indicating the maximum distance from the mannequin. The procedure from the ready period to the reporting period counted as one trial, with the task comprising 25 trials in total. The five spider stimuli used in the fear rating task were presented once at each of the five vertical locations (closest, second closest, middle, second farthest, or farthest) in random order (i.e., five trials at each location).

For the data analysis, we fitted a linear regression to individuals’ estimated values, assigning numbers from -2 to 2 to the five stimulus locations (-2: farthest, -1: second farthest, 0: middle, 1: second closest, and 2: closest). The regression analysis revealed three participants reported estimated values failing to demonstrate a significant linear increase from nearest to farthest locations (*p* > 0.05). We excluded their data from the analyses because these participants did not perceive interval lengths across five stimulus locations as equal.

**2. Inclusion of initial trials of each block**

Although the initial trials of each block were excluded from the analysis because their potential prominence might affect the summary of the collected data, we also analyzed the fear ratings in Experiment 1 by including the discarded initial trials. Note that the initial trials of each block (two trials per run, eight trials in total) were planned in advance to be excluded from the main analysis, and their stimulus type and location were randomly selected. First, we applied a robust linear mixed-effects model to the fear ratings for the spider stimuli, revealing a significant main effects of both visuotactile congruency (*β* = 0.053, *SE* = 0.024, *t*_117_ = 2.23, *p* = 0.028) and stimulus location (*β* = 0.16, *SE* = 0.024, *t*_117_ = 6.60, *p* < 0.001), but the interaction between these two factors did not reach significance (*β* = 0.040, *SE* = 0.024, *t*_117_ = 1.69, *p* = 0.093). Next, the difference in the fear ratings between the proximal and distal spider stimuli was significantly larger in the congruent condition than in the incongruent condition (*Z* = 2.06, *p* = 0.040, *ρ*_rb_ = 0.41).

Regarding the fear ratings for the butterfly stimuli, a robust linear mixed-effects model revealed a significant main effect of stimulus location (*β* = 0.019, *SE* = 0.0090, *t*_117_ = 2.07, *p* = 0.041). In contrast, neither the main effect of visuotactile congruency (*β* = -0.0091, *SE* = 0.0090, *t*_117_ = -1.01, *p* = 0.31) nor the interaction (*β* = -0.0013, *SE* = 0.0090, *t*_117_ = -0.14, *p* = 0.89) was significant. The proximal-distal difference did not significantly differ between the visuotactile congruency conditions (*Z* = 0.75, *p* = 0.47, *ρ*_rb_ = 0.19).

Even with the initial trials of each block included, our findings indicate that subjective fear remains sensitive to stimulus location during the body ownership illusion. However, a novelty effect and a random selection of the initial trial stimuli probably weakened the interaction between visuotactile congruency and stimulus location for the spider stimuli and reduced the effect size for the main effect of stimulus location in both spider and butterfly stimuli.

**Supplementary Figures**


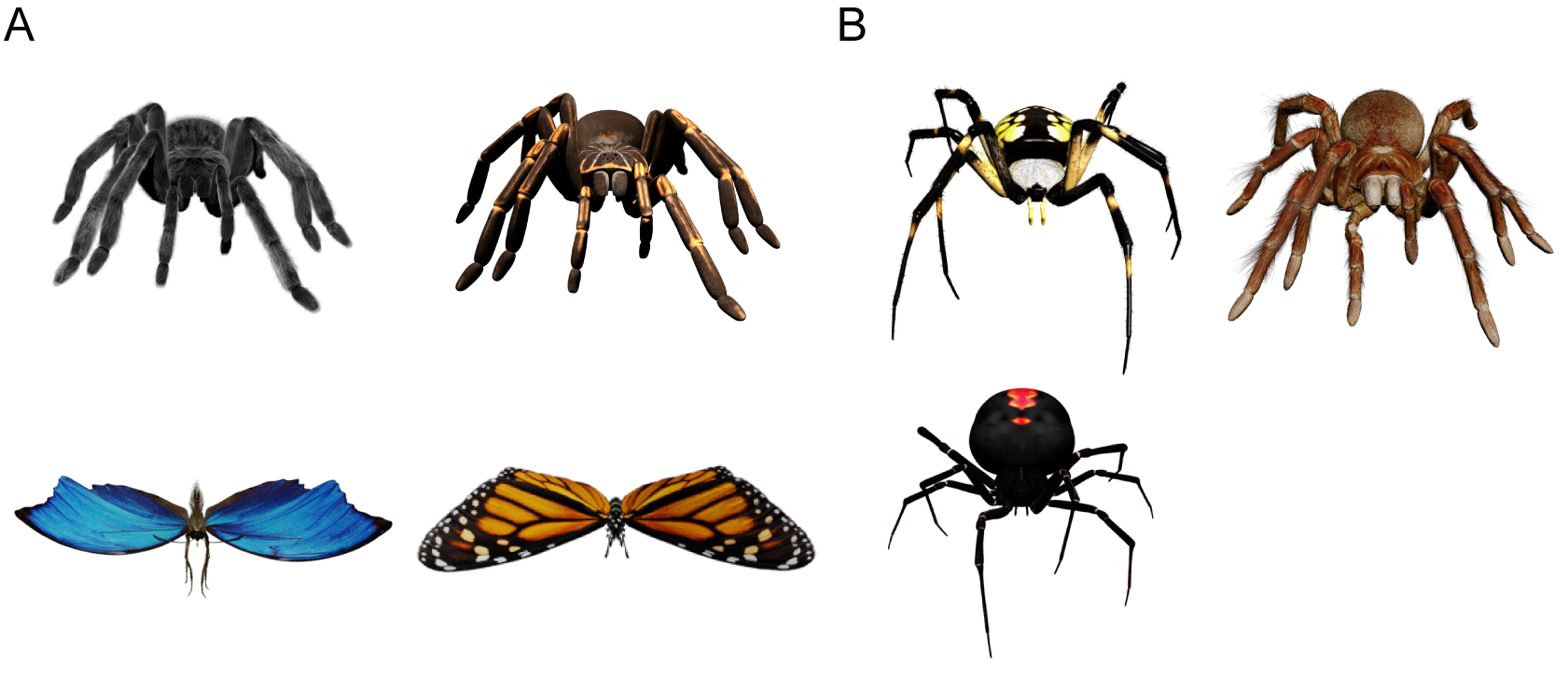


**Supplementary Fig. 1 | 3D spider and butterfly animations. A** Rendered images of 3D spider and butterfly animations used in Experiment 1. Two different types of spiders and two different types of butterflies were selected from the 3D model collection site. To create fluid movement in the task video, 90 rendered images were generated for each animation and were presented sequentially over a 3-second stimulus period (see also Supplementary Videos 1, 2, and 3). **B** Rendered images of additional 3D spider animations used in Experiment 2. Three new types of spiders were selected from the 3D model collection site.

**
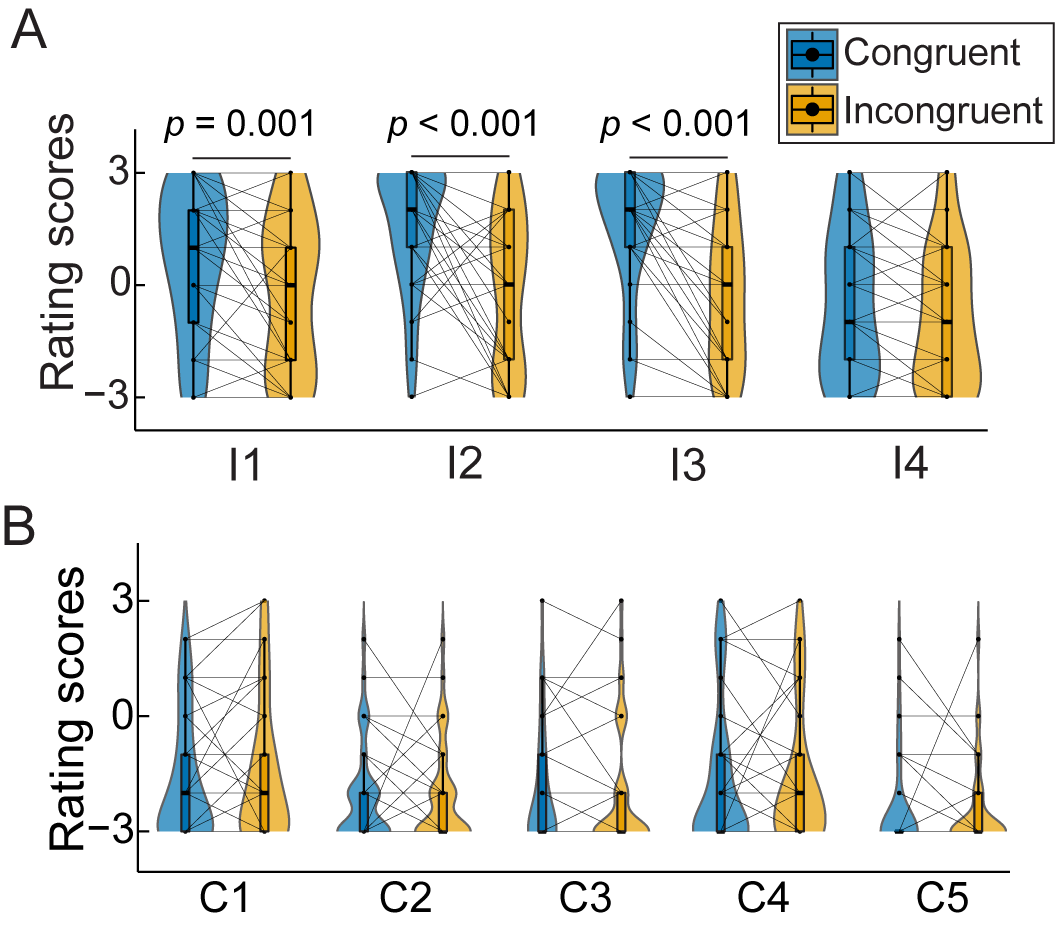
**

**Supplementary Fig. 2 | Rating scores for individual statements in the illusion-assessment task in Experiment 1. A** The rating scores for illusion statements (I1–I4). **B** The rating scores for control statements (C1–C5). In each box plot, the central horizontal line indicates the median, while the bottom and top edges of the box indicate the 25th and 75th percentiles, respectively. The whiskers of each box plot extend 1.5 times the interquartile range from each hinge. Each dot represents an individual participant.

**
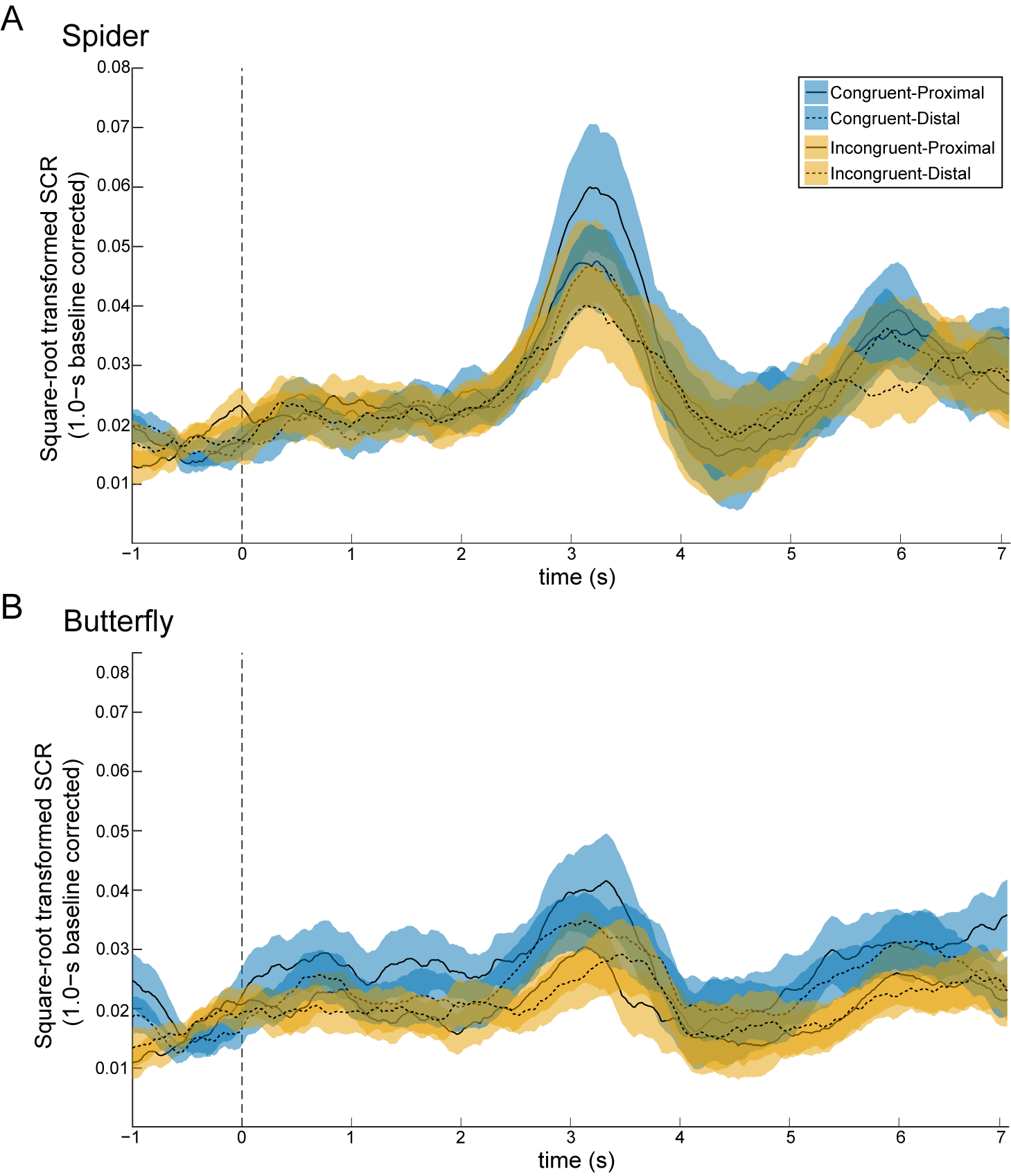
**

**Supplementary Fig. 3 |** **Time courses of SCR in Experiment 1. A** Time courses of SCR to spider stimuli. **B** Time courses of SCR to butterfly stimuli. Solid lines represent SCRs to proximal stimuli, while dashed lines indicate SCRs to distal stimuli. The two visuotactile congruency conditions were separately plotted, blue-green and orange representing the Congruent and Incongruent conditions, respectively. The time courses were baseline-corrected using data from 1 s before the stimulus presentation and then square-root transformed for normalization. Colored shaded areas denote standard errors.

**
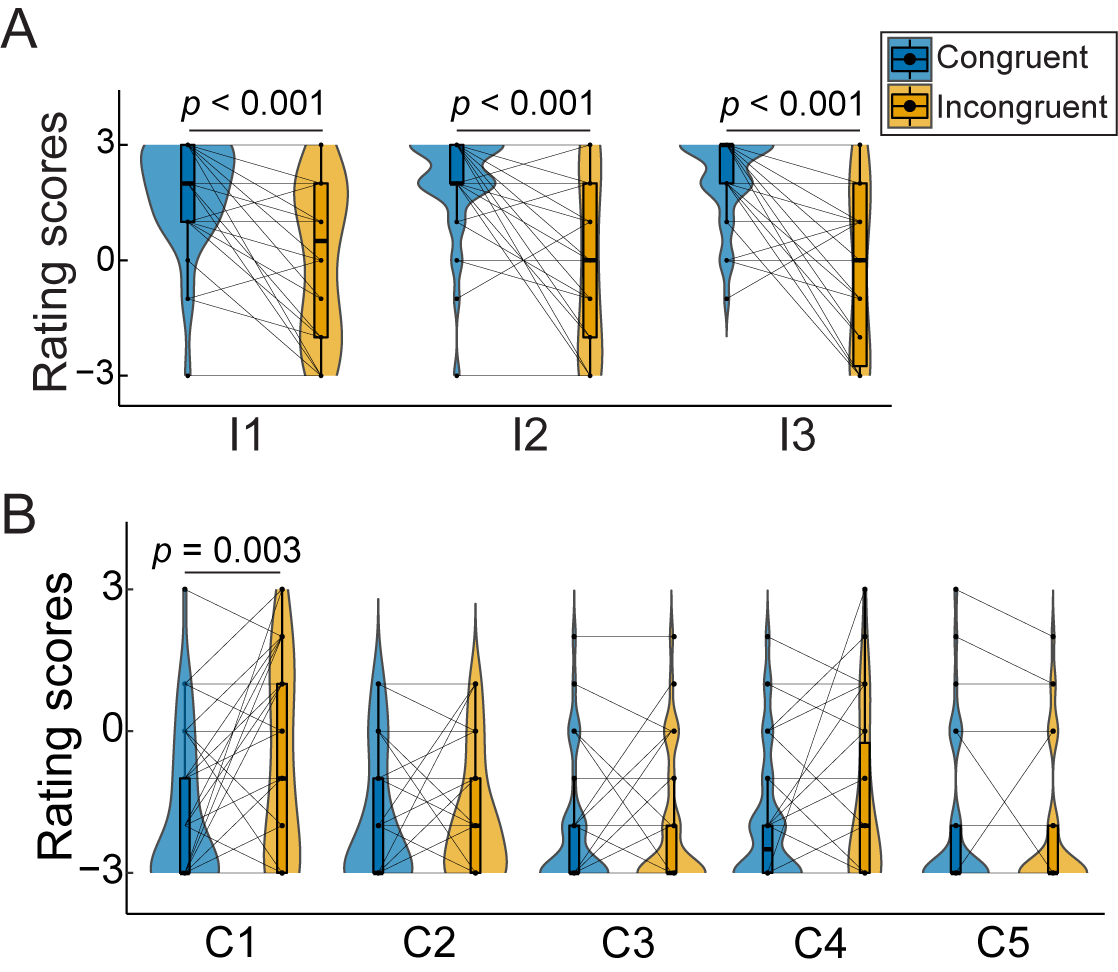
**

**Supplementary Fig. 4 | Rating scores for individual statements in the illusion-assessment task in Experiment 2.** **A** The rating scores for illusion statements (I1–I3). **B** The rating scores for control statements (C1–C5). In each box plot, the central horizontal line indicates the median, while the bottom and top edges of the box indicate the 25th and 75th percentiles, respectively. The whiskers of each box plot extend 1.5 times the interquartile range from each hinge. Each dot represents an individual participant.

**Captions for Supplementary Videos**

**Supplementary Video 1 | Example of proximal spider stimulus for experimental participants.**

An example trial in the fear rating task of Experiment 1. A 3D spider stimulus is presented at the proximal location relative to a mannequin. A trial includes the stimulus and rating and short stroking periods.

**Supplementary Video 2 | Example of distal spider stimulus for experimental participants.**

An example trial in the fear rating task of Experiment 1. A 3D spider stimulus is presented at the distal location relative to a mannequin.

**Supplementary Video 3 | Example of proximal butterfly stimulus for experimental participants.**

An example trial in the fear rating task of Experiment 1. A 3D butterfly stimulus is presented at the proximal location relative to a mannequin.
